# Incidental Diagnosis of Cholecystocolonic Fistula in An Asymptomatic Male: A Rare Case Presentation

**DOI:** 10.7759/cureus.101933

**Published:** 2026-01-20

**Authors:** Navdeep Bais, Kara Harris, Sanjiv Bais, Syed U Hamdani

**Affiliations:** 1 Medicine, Ross University School of Medicine, Bridgetown, BRB; 2 General Surgery, St. Vincent Medical Center, Toledo, USA; 3 General Surgery, Mercy Health - St. Anne Hospital, Toledo, USA; 4 Gastroenterology, Mercy Health - St. Anne Hospital, Toledo, USA

**Keywords:** cholecystocolonic fistula (ccf), cholecystoenteric fistula, colon resection, robotic colorectal surgery, screening colonoscopy

## Abstract

Cholecystocolonic fistula (CCF) is a rare complication of chronic gallbladder disease, most commonly seen in elderly women aged 60-70 years. Although most cases are detected on imaging, some are discovered incidentally during surgery. If discovered preoperatively, management can be provided operatively or nonoperatively, depending on symptoms and severity. We present a case of a CCF that was discovered in an asymptomatic 72-year-old man during routine colonoscopy screening. Imaging confirmed the diagnosis, and surgical management was performed. This was initially attempted robotically, but was converted to an open cholecystectomy with a proximal transverse colon resection. The patient's postoperative course was unremarkable, and was followed up for a three-month period after their surgery. This case highlights the atypical presentation of CCF and current diagnostic challenges and surgical approaches.

## Introduction

Cholecystocolonic fistula (CCF) is an abnormal connection between the gallbladder and the colon, most commonly occurring in the region of the hepatic flexure, due to their close anatomical proximity. Development of these fistulae typically results from chronic gallbladder disease and recurrent episodes of cholecystitis that ultimately lead to necrosis and breakdown of the gallbladder wall into the adjacent colonic wall [1].

CCFs account for approximately 8% of all cholecystoenteric fistulas and are most often seen in women who are in the age group of 60-70 years [1-3]. They occur in approximately 0.06-0.14% of patients with gallbladder disease, making them a rare biliary pathology [1]. While often asymptomatic, symptomatic patients may have a classic triad of chronic diarrhea, fat-soluble vitamin malabsorption, and pneumobilia [2].

Preoperative diagnosis of CCF can be a challenge, as no single imaging modality has proven to be a reliable diagnostic method. These fistulas are usually discovered incidentally in approximately 0.5% of cholecystectomy cases [1]. Although there are no clear guidelines for CCF management, surgical management typically involving cholecystectomy and fistula tract excision is generally considered the treatment of choice [3,4]. This case report examines an incidental finding of a CCF during a routine screening colonoscopy in an elderly, asymptomatic male patient and subsequent management. It emphasizes the importance of clinician awareness in recognizing this rare pathology and determining appropriate further management.

## Case presentation

A 72-year-old male patient presented for a routine screening colonoscopy. He had a history of one screening colonoscopy approximately 11 years ago, which was normal. Prior to the procedure, the patient did not report any bloody stool, changes to bowel habits, frequency, abdominal pain, or weight loss. 

**Figure 1:**
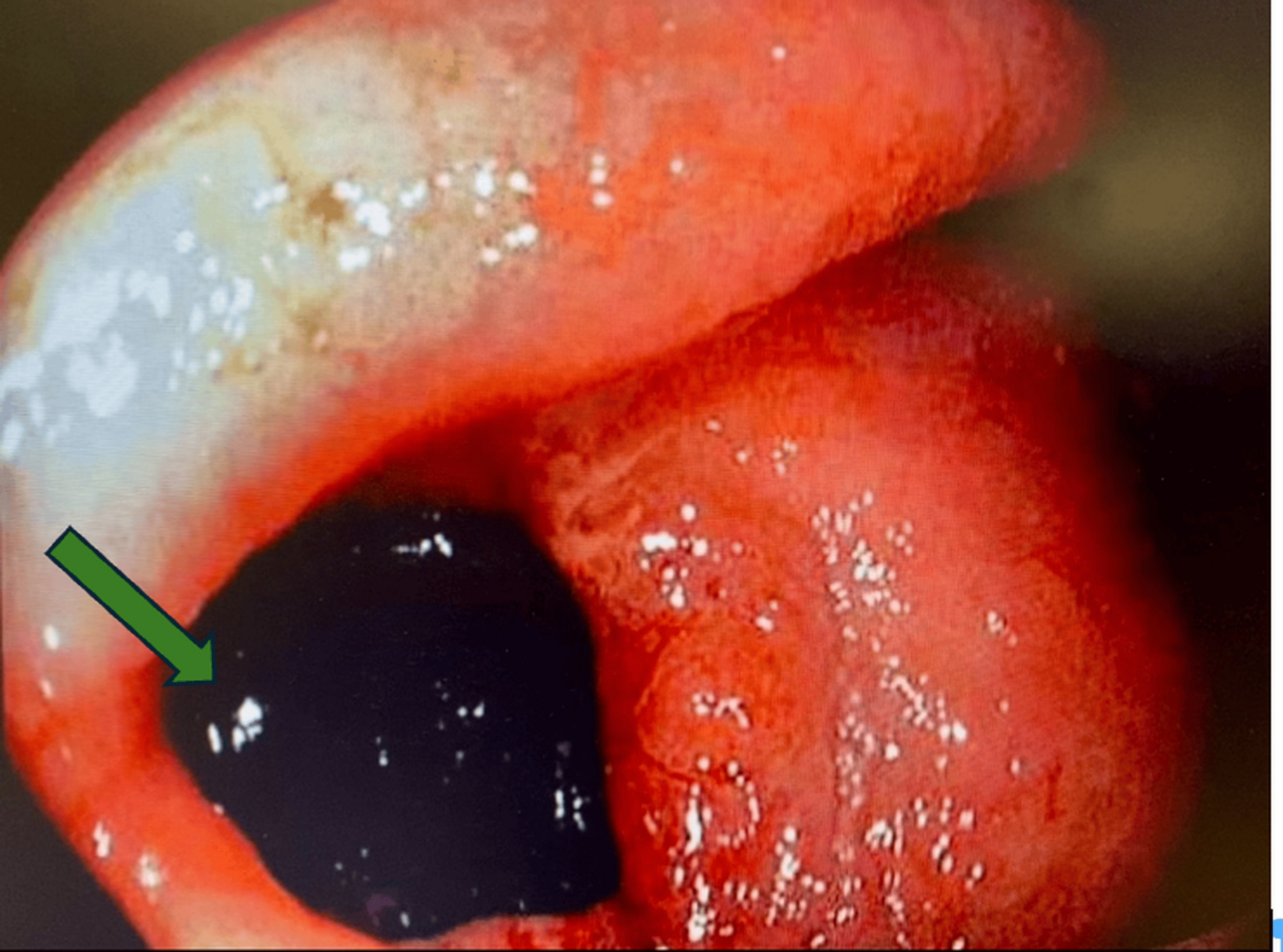
Endoscopic view showing gallstones (green arrow) in the fistula tract.

Colonoscopy revealed six polyps ranging from 4 mm to 6 mm in the ascending colon, a 20 mm inflammatory mass at the hepatic flexure, which resembled a diverticular opening, a few small diverticula in the descending/sigmoid colon, a 5 mm sigmoid colon polyp, and large internal hemorrhoids. Biopsies of the granulation tissue at the hepatic flexure demonstrated inflammatory changes. Due to these findings, a repeat colonoscopy was done and resulted in a diagnosis of a CCF at the hepatic flexure. Gallstones were present inside the fistula (Figure 1). 

**Figure 2:**
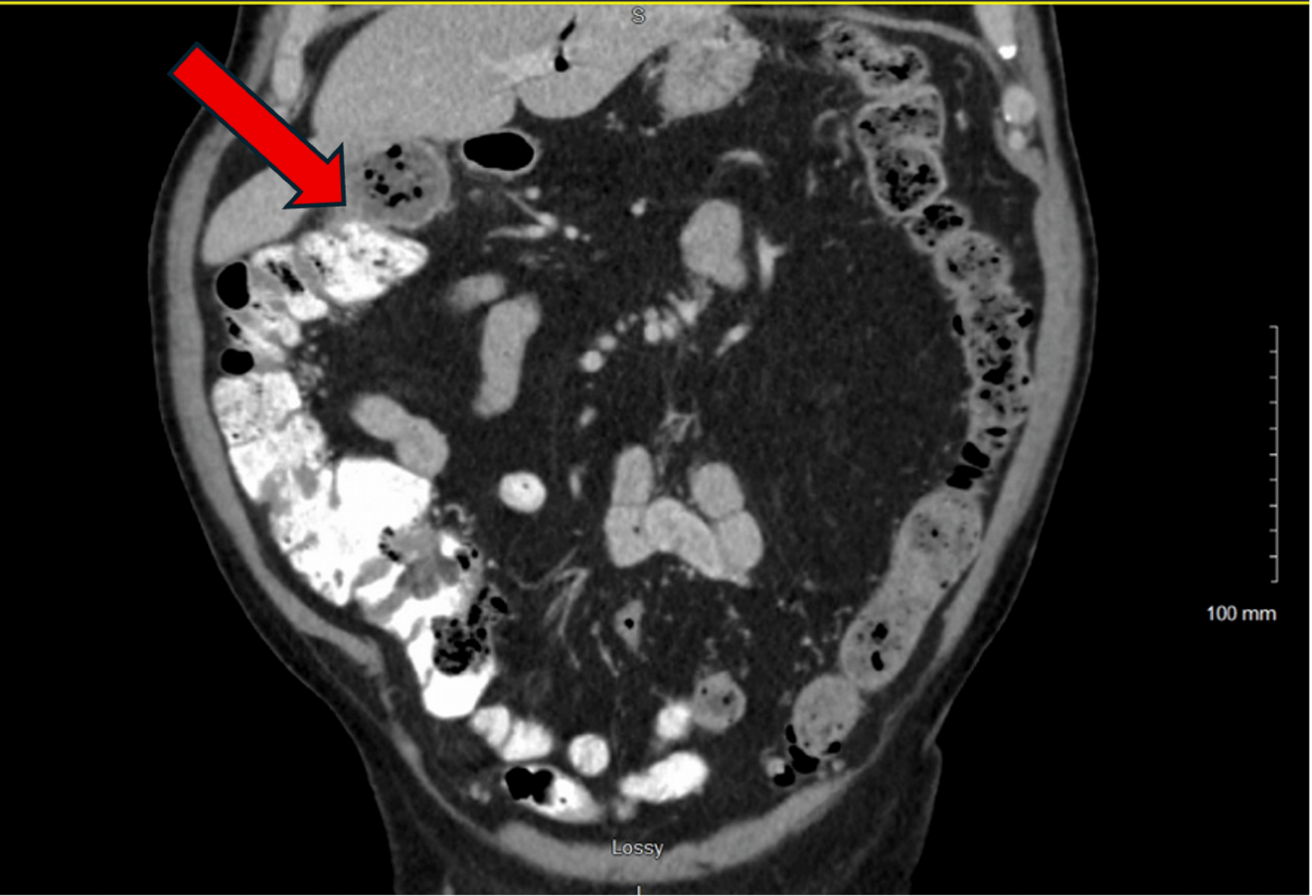
Coronal computed tomography image with intravenous contrast demonstrating a cholecystocolonic fistula in the region of the hepatic flexure.

**Figure 3:**
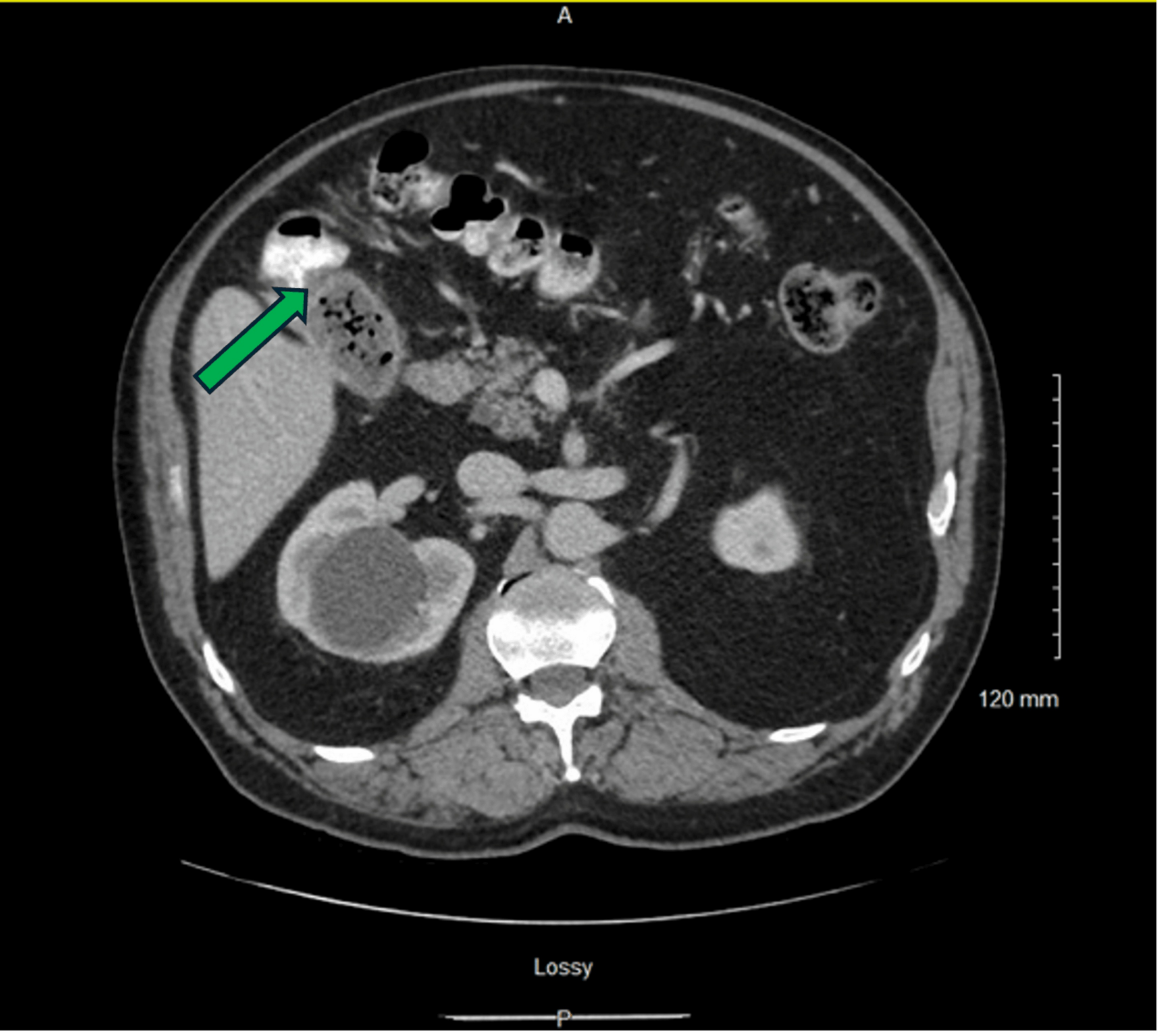
Axial computed tomography image with intravenous contrast demonstrating the fistula with associated pneumobilia.

**Figure 4:**
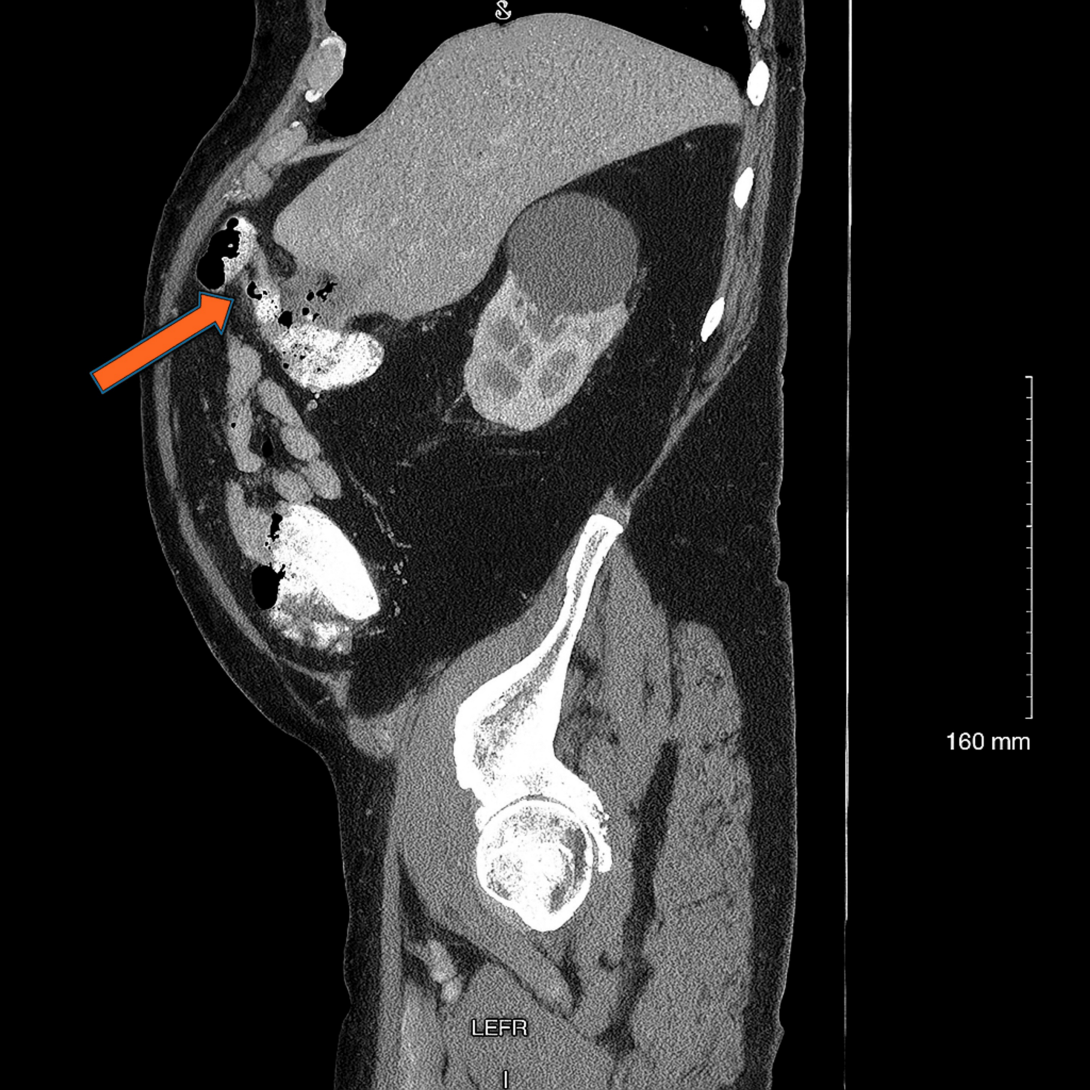
Sagittal computed tomography image with intravenous contrast providing an additional view of the fistula and pneumobilia.

After CT confirmation of the fistula (Figures 2-4), surgical intervention was performed. The initial operative plan was for a robotic cholecystectomy with segmental resection of the involved bowel and fistula. Robotic mobilization of the ascending colon, hepatic flexure, and mid-transverse colon was performed, along with lysis of adhesions, which likely formed due to the patient's history of an appendectomy. It was difficult to separate the transverse colon from the gallbladder due to extensive inflammatory processes, leading to poor visualization and indetermination of the anatomy. Consequently, the decision to convert this to an open procedure was made.

A right subcostal incision was performed, as the colon had already been mobilized robotically. The bowel was then transected proximally and distally to the fistula in the transverse colon. The gall bladder was quite large and firm upon palpation. A retrograde cholecystectomy was performed, along with en bloc resection of the transverse colon, which also incorporated the fistula. Approximately 7 cm of the transverse colon was resected, and a stapled side-to-side anastomosis was created of the remaining transverse colon segments. The pathology report showed that the collected specimen consisted of gallbladder and transverse colon, and the gallbladder was described as gallbladder with chronic cholecystitis. The surgical margins were benign, and the specimen was negative for dysplasia and malignancy. 

Postoperatively, the patient remained in the hospital for three days. Bowel function returned on day two. A Jackson-Pratt drain that was placed intraoperatively in the liver bed for hemostasis and drainage produced serosanguinous fluid, with decreasing outputs reported daily. This drain was removed before discharge on postoperative day three. No complications were reported, and the patient was discharged in stable condition. The patient was subsequently followed up in the clinic for three months and had an unremarkable postoperative recovery.

## Discussion

CCFs are a rare sequela of chronic gallstone disease, typically resulting from repetitive bouts of cholecystitis leading to necrosis and erosion of the gallbladder wall into the colonic wall, typically at the hepatic flexure [1]. The first documented case of a CCF was described in 1890 by Courvoisier [5]. CCFs account for approximately 8% of all cholecystoenteric fistulas, and occur in approximately 0.06-0.14% of patients with any form of gallbladder disease [1,6]. The typical demographic that these fistulas are seen in is usually women aged 60-70 [1,2]. This makes our patient, an asymptomatic 72-year-old man, an atypical epidemiological presentation. 

Though CCFs are often asymptomatic and incidentally detected, symptomatic patients usually present with a triad of vitamin malabsorption, pneumobilia, and chronic diarrhea (secondary to bile acid malabsorption) [7]. Our patient did not exhibit any of these symptoms. Although these fistulas are a rarity, this abnormal connection can lead to many serious complications, including life-threatening biliary sepsis, migration of biliary fluids, enteric bacterial translocation, liver abscesses, and colonic obstruction secondary to gallstone migration [6,8]. 

Preoperative diagnosis of CCF remains a challenge. Krzeczowski et al. reported that approximately 8% of CCF diagnoses are made preoperatively [9]. A wide array of imaging modalities can be used for a preoperative diagnosis, including CT scan, MRI, ultrasound, and endoscopic retrograde cholangiopancreatography (ERCP), but no one modality has been proven to be highly sensitive or superior to others [2,6,7]. Ultrasound, which is usually the first modality used for suspected hepato-biliary pathologies, has not proven to be a reliable source of diagnosis for CCF. Invasive methods, such as ERCP and colonoscopy, have been explored, but the results of using these for diagnosis have been inconsistent. Our literature search revealed no documented cases of a CCF diagnosis being made during routine colonoscopy screening, which makes this case a unique presentation. Literature has shown the diagnosis of CCF being made in symptomatic patients who have undergone a for-cause colonoscopy. Hens and Reynaert report the diagnosis of a CCF in a patient who underwent a colonoscopy after chronic diarrhea and weight loss over a three-month period [4]. Bonventre et al. describe a diagnosis of CCF being made through colonoscopy in a patient with a fever of unknown origin for approximately two months [10]. Of note, CCF diagnoses are made intraoperatively at a rate of approximately 0.5% [1]. 

There are no clear guidelines for the management of CCF, but per the literature, management typically involves cholecystectomy and fistula tract excision [3,4]. This differs from our case, as we had to resort to a segmental resection of the transverse colon en bloc with the gallbladder, as dense attachments between the gallbladder and the colon made it difficult to separate them from each other.

Prior to the 1990s, a laparoscopic approach to this procedure was contraindicated, with an open approach being preferred, but since then, laparoscopic procedures have widely adopted due to decreased morbidity and improved recovery [6,11]. Quite frequently, a conversion to an open procedure is necessitated due to adhesions being present secondary to chronic inflammation. Angrisani et al, in a multicenter study published in 2001, reported that a conversion rate to an open procedure was necessary in approximately 55% of cases [7]. In our case, an initial robotic attempt was converted to an open procedure due to poor visualization secondary to inflammation between the gallbladder and the colon.

Robotic-assisted surgeries offer enhanced visualization, decreased morbidity, and faster recovery times for patients. However, there is limited literature available for CCFs managed robotically. Krzeczowski et al. discuss a robotic approach in a 79-year-old male patient after a diagnosis was made from a CT scan and ERCP [9]. Another case discusses a 50-year-old woman with a diagnosis of CCF approximately five years after the patient was diagnosed with cholangitis [8]. This patient subsequently had robotic surgery and made a complete recovery. These cases suggest the benefits of robotic-assisted surgery, but larger studies are lacking. 

## Conclusions

CCF is a rare complication of chronic gallbladder disease, often seen in elderly female patients. This case highlights an uncommon presentation of CCF: an asymptomatic 72-year-old man who had CCF discovered incidentally during routine colonoscopy screening. The rarity of this presentation emphasizes the need for awareness of atypical presentations when evaluating a patient and thinking of differential diagnoses. Surgical intervention and correction remain the standard of care, with different approaches tailored to anatomical variations and intraoperative findings. This case highlights the fact that clinicians should think broadly with their differential diagnoses and should also consider rare pathologies when evaluating a patient with inconclusive colonic or biliary findings.
